# Machine learning approach to literature mining for the genetics of complex diseases

**DOI:** 10.1093/database/baz124

**Published:** 2019-11-26

**Authors:** Jessica Schuster, Michael Superdock, Anthony Agudelo, Paul Stey, James Padbury, Indra Neil Sarkar, Alper Uzun

**Affiliations:** 1 Department of Pediatrics, Warren Alpert Medical School of Brown University, Providence, RI, 02903, USA; 2 Department of Pediatrics, Women & Infants Hospital of Rhode Island, Providence, RI, 02905, USA; 3 Computing and Information Services, Brown University, Providence, RI, 02903, USA; 4 Center for Biomedical Informatics, Brown University, Providence, RI, 02912, USA; 5 Center for Computational Molecular Biology, Brown University, Providence, RI, 02906, USA; 6 Rhode Island Quality Institute, Providence, RI, 02908, USA

## Abstract

To generate a parsimonious gene set for understanding the mechanisms underlying complex diseases, we reasoned it was necessary to combine the curation of public literature, review of experimental databases and interpolation of pathway-associated genes. Using this strategy, we previously built the following two databases for reproductive disorders: The Database for Preterm Birth (dbPTB) and The Database for Preeclampsia (dbPEC). The completeness and accuracy of these databases is essential for supporting our understanding of these complex conditions. Given the exponential increase in biomedical literature, it is becoming increasingly difficult to manually maintain these databases. Using our curated databases as reference data sets, we implemented a machine learning-based approach to optimize article selection for manual curation. We used logistic regression, random forests and neural networks as our machine learning algorithms to classify articles. We examined features derived from abstract text, annotations and metadata that we hypothesized would best classify articles with genetically relevant content associated to the disorder of interest. Combinations of these features were used build the classifiers and the performance of these feature sets were compared to a standard ‘Bag-of-Words’. Several combinations of these genetic based feature sets outperformed ‘Bag-of-Words’ at a threshold such that 95% of the curated gene set obtained from the original manual curation of all articles were extracted from the articles classified by machine learning as ‘considered’. The performance was superior in terms of the reduction of required manual curation and two measures of the harmonic mean of precision and recall. The reduction in workload ranged from 0.814 to 0.846 for the dbPTB and 0.301 to 0.371 for the dbPEC. Additionally, a database of metadata and annotations is generated which allows for rapid query of individual features. Our results demonstrate that machine learning algorithms can identify articles with relevant data for databases of genes associated with complex diseases.

## Introduction

To better understand the genetic mechanisms of complex diseases, we generated a manageable set of biologically validated genes that incorporates the elements of the discovery in genome-wide investigations. Our strategy was to identify relevant, phenotype-specific gene sets that combined the curation of public literature, review of experimental databases and interpolation of pathway-associated genes. We used web-based semantic data mining of published literature to recover articles that contained genes or genetic variants potentially related to diseases of interest. To add a *discovery-based* approach to our strategy, we also screened publicly available, genome-wide databases for additional information. Curators read each article and identified the genes supported by experimentally validated biological relevance for the conditions of interest. Using this strategy, we built publicly available databases for two complex reproductive disorders: (i) the Database for Preterm birth (dbPTB) and (ii) The Database for Preeclampsia (dbPEC) (1, 2).

The completeness and accuracy of genetic databases is essential for our understanding of complex disease phenotypes. These databases serve not only as a concise collection of past genetic findings, but also the foundation upon which many new gene-disease discoveries are made (3, 4). Unfortunately, given exponential increases in biomedical literature, it is increasingly difficult to maintain such databases manually (5–8). Manual curation is inevitably outpaced by literature production (6, 9). Even so, manually verified data remain the gold standard for genetic databases. This demand has motivated the development and implementation of computational tools to automate or semi-automate various steps of the biocuration workflow including literature screening or ‘triage’, bioentity identification, relationship annotation and data normalization, all with the goal of minimize the curator workload without sacrificing accuracy (10–16).

The first step in the curation workflow once literature is queried is often referred to as ‘triage.’ Triage involves identifying abstracts as candidates for full curation and further data extraction (14, 17). It is of great interest to support this process using automated means, as it often one of the largest bottlenecks and subject to human error (14, 17, 18). The first step in automating screening involves text mining the title and abstract. Many text-mining tools have helped automate some of the more basic tasks of curation, such as identifying mentions of biological entities in free text. PubTator is one example. This ensemble program combines the functionality of several other entity recognition tools, including GeneTUKit (28) for gene mentions, SR4GN (29) for species, DNorm (30) for diseases and tmVar (31) for mutations, thereby allowing curators and other text-mining tools to extract these entities more efficiently. Another commonly used entity recognition tool, MetaMap, recognizes mentions of a wide variety of entities and maps them to Unified Medical Language System (UMLS) concepts. SemRep further extends the functionality of MetaMap by using these UMLS concepts to identify subject-predicate-object triples. Texpresso is another popular text-mining tool, which utilizes ontology bases categories for information retrieval and data extraction, developed for the model organism *Caenorhabditis elegans* and currently expanding to other models (19).

These text-mining tools can be used in conjunction with classification, ranking or active learning to reduce the number of documents that must be manually screened. The number of studies devoted to this endeavor is increasing; however, there is little consensus as to best approach. As noted, many are not fully documented, have limited evaluation of performance, and are not freely accessible (20). Additionally, results are difficult to reproduce as they are dependent on the complexity of research question being studied, the heterogeneity of the literature, the size of literature base, and the consistency of the curation team. Wormbase, one of the most comprehensive gene-centric database about *C. elegans* implements support vector machine classifiers and Textpresso for flagging data types within documents (19); however, the first step of screening papers for further curation and data flagging and extraction is preformed manually (21). Other tools, such as AbstrackR were designed for semi-automating systematic reviews using machine learning in the absence of any entity recognition or literature annotating bag-of-words (unigrams and bigrams) as features of support vector machine classifiers (13). This tool has been shown to have high recall, with mixed results for precision and other evaluation metrics (20, 22).

In this extension of prior work, we designed a method that allows curators to partially automate the literature review for identifying articles related with genetic data associated to the phenotype of interest for subsequent manual curation and data extraction. We used the previously developed databases as reference sets for testing different models of machine learning. The machine learning algorithms evaluated for this study were logistic regression, random forests and neural networks. Logistic regression seeks to identify a linear combination of variable coefficients that best estimates the probability that an article should be considered (23). Random forests uses a collection of individual decision trees and the mean outcome generates a probabilistic estimation that an article should be considered (24). Artificial neural networks uses an interconnected group of computing nodes that ‘fire’ when the weighted sum of inputs to the node is sufficiently large. Weights are learned so that features of an article produce output with signal intensity that represents the probability that the article should be considered (25). The standard features used for machine learning are ‘Bag-of-Words’ or variations of such. We built the classifiers with multiple features, which we hypothesized would better identify genetic relevance. We compared how these individual features, as well as combination, performed when compared to a standard ‘Bag-of-Words’ feature set. Our approach relied on automated text-mining tools to extract rich features from titles and abstracts, supervised machine learning to predict article relevance and human expertise to identify relevant genetic data.

## Methods

### dbPTB and dbPEC

In building these two databases, *SciMiner^Tm^* (26) was used to identify articles from PubMed using a number of queries to extract published articles specific to preterm birth and preeclampsia and their gene and protein information (1, 2). The queries used for each database are listed in Supplementary Table S1. The filtered articles putatively contained information on genes, gene–gene interactions, or single-nucleotide polymorphism (SNP) information related to preterm birth or preeclampsia. A curation team member then read each publication, with attention devoted to study design, relevance of the article to the phenotype of interest, and documentation of statistical relationship between a gene and either preterm birth or preeclampsia. Articles with at least one relevant gene were labeled as ‘considered’ for curation; those with no relevant genes were labeled as ‘not considered.’ The genes, genetic variants, SNPs, Reference SNP (rs) numbers (when available) and annotations describing gene–gene interactions shown to be statistically significant from each considered article were entered into the databases.

### Training and test sets

**Figure 1:**
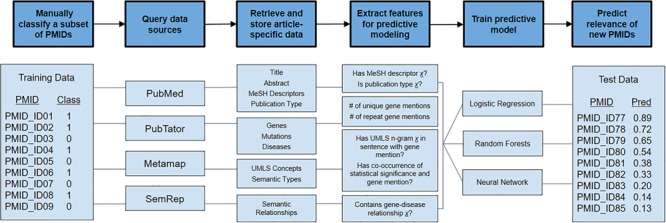
Overview of semi-automated pipeline used to predict articles to consider for manual curation. The pipeline takes a set of PMIDs (here shown as PMID_ID##) as input along with manual classifications (class) of 1 or 0, where 1 signifies that an article was ‘considered’ for curation and 0 signifies that an article was ‘not considered’. With these PMIDs, the computational pipeline queries various public data repositories to retrieve article-specific data. Data are converted to features useful for predictive modeling. Using this feature set, predictive models were trained using logistic regression, random forests and neural networks. These predictive models were used to predict the relevance of unread articles.

We accessed all articles and the genes that had been reviewed by manual curation for the development of dbPEC and dbPTB (1, 2). The records included 2667 articles curated for dbPEC and 1530 articles curated for dbPTB. We created training and tests sets for both dbPTB and dbPEC independently. For the training sets, 80% of the PubMed Identifiers (PMIDs) were randomly selected from both the set of considered and not considered articles for dbPEC and dbPTB. Samples were randomly selected using a program written in the Julia programming language (https://epubs.siam.org/doi/10.1137/141000671), leveraging the Mersenne Twister library to create a random number generator and using it to sample from the set articles. This random selection was carried out to mimic the accepted and rejected rates of the dbPEC and dbPTB. The remaining 20% of the considered and not considered articles were used to validate our model. PMIDs and manual classifications for articles were used as input into our computational pipeline, which was designed to retrieve article metadata and annotations, generate features for each article, and train predictive models in order to prioritize unseen articles for curation (Figure 1).

### Data retrieval

For each PMID in our training set, data were retrieved from four sources: PubMed/MEDLINE, PubTator (27), MetaMap (28) and SemRep (29) . PubMed was used to retrieve the title, abstract and metadata for each article. PubTator, MetaMap, and SemRep were used as sources for annotating titles and abstracts. PubTator was used to annotate genes, diseases, species and mutations. MetaMap was used to identify UMLS mappings, while SemRep was used to identify relationships between different biological concepts in our articles. The information from these data sources were processed and stored in a local MySQL database. Details for querying and processing data from each of these data sources are detailed in the Supplemental Materials.

### Feature generation

Document data stored in the local MySQL database was used to generate features for input into a machine learning algorithm. Many of the features generated utilized UMLS concepts grouped by similar semantic type (‘semtype’). The features used in the predictive models were generated according to the specifications below.

### Bag-of-Words

‘Bag-of-Words’ was used as a baseline method to compare the performance of feature sets. Words in the title and abstract were tokenized and the frequency of the unigrams was recorded. The corpus was stripped of indefinite articles, definite articles, prepositions, pronouns and stop words. Words were also appended to have either the TITLE or ABSTRACT suffix.

### Medical subject headings

Medical Subject Headings (MeSH) heading-subheading-major triplets were generated as a feature set. For example, an article with the MeSH descriptor ‘Premature Birth’ may have the associated descriptor ‘Genetics’, where ‘Genetics’ is labeled as a major focus of the article but ‘Premature Birth’ is not. One feature is generated for each heading and subheading. The heading-subheading-major feature for ‘Premature Birth’ is *Premature Birth-No*. The heading-subheading-major feature for ‘Genetics’ is *Premature Birth-Genetics-Yes*.

### Gene-subject-predicate

SemRep annotations were used to create the subject-relation-object feature set. Features were generated for each sentence for which the subject was in the semantic group ‘GENE’ (genes and molecular sequences) and the object was in the semantic group ‘DISO’ (disorders). In addition features were generated for sentences where the subject was in the semantic group ‘DISO’ and the object was in the semantic group ‘GENE’.

### Gene and statistical significance co-occurrence

UMLS concepts were used to identify sentences with Quantitative Concepts (‘qnco’) to generate the (Quantitative CUI)-(Gene Presence) feature set. A feature was generated for a sequence which contained a unique Quantitative Concept with an indicator for co-occurrence with the semantic group ‘GENE’.

In addition, UMLS concepts were used to identify sentences in which there was an occurrence of the concepts related to statistical significance. The UMLS Terminology Services (UTS) Metathesaurus Browser was used to search for concepts related to statistical significance, *P* value, and z-score; these concepts were called ‘significant Concept Unique Identifiers (CUIs)’. In addition, sentences in which there was a co-occurrence between concepts in the ‘GENE’ semantic group and ‘significant CUIs’ were identified and features were appended with either having a gene present or not. With each occurrence of ‘significant CUIs’, the presence of the UMLS concept ‘Negative’, ‘Negation’ or ‘Unchanged’ was also identified. This was done to handle cases where genetic data were described as *not* statistically significant. Features were generated as triples: (significant cui)-(GENE presence)-(Negation).

### Gene counts

PubTator was used to identify and create the gene count feature set for both the title and the abstract. Features were generated for the number of unique gene names referenced in the title and the abstract. Features were also generated for the maximum number of references to a single gene in the abstract and title respectively.

### Species check

In the two reference databases, articles were only considered if human tissues or cells were being studied. UMLS Concepts were used to identify the species present in the abstract and create the (Species CUI)-(GENE Presence) feature set. Features were generated for the sentences with semantic types in the ‘LIVB’ semantic group with an indicator for co-occurrence with the semantic group ‘GENE’.

### Semtype count

To reduce feature size and allow for generalizability, UMLS concepts were used to identify generic groups of similar concepts and create the (semantic type)-(GENE-Present) feature set. Features were created for each semantic type with an indicator for co-occurrence with the ‘GENE’ semantic group.

## Machine learning

### Predictive models

We employed three machine learning algorithms to compare their ability to predict which articles should be considered for manual curation: (i) logistic regression, (ii) random forests and (iii) neural networks. For each of the machine learning algorithms, articles are assigned a predictive probability between 0 and 1 where an article close to a predictive probability of 1 is likely to be considered for curation and an article closer to 0 is unlikely to be considered for curation.

### Parameter optimization

The logistic regression, random forest, and neural network classifiers were implemented using Scikit-learn (30). The hyper-parameters for the classifiers were optimized using randomized search cross-validation (31). The *Semtype Count* feature set was used to optimize the hyper-parameters for the various classifiers.

Studies have shown (32) that when tuning hyper-parameters for random forest that the max_features, min_sample_split, and min_sample_leaf, hyper-parameters have the largest effect on prediction accuracy. When tuning for Logistic Regression hyper-parameters, three hyper-parameters were optimized for max_iter, C and solver (33). Neural Networks were optimized for hidden_layer_sizes, learning_rate (learning rate schedule), alpha and learning_rate_init (31). A more in-depth explanation of the different hyper-parameters that were optimized for each of the classifiers is given in the Supplemental Materials.

### Class imbalance

In both data sets, there was an imbalance in accepted and rejected papers. Of the 2667 articles related to preeclampsia, 898 articles (33.7%) were considered to contain relevant information following manual curation. Of the 1530 articles related to preterm birth, only 204 articles (13.3%) were considered to contain relevant information following manual curation. To address class imbalance, under-sampling, oversampling and a combination of the two were evaluated, as well as assigning class weights inversely proportional to class frequencies. This was done for all three predictive models. These predictive models were trained against training set articles and evaluated for accuracy against model-naïve test sets.

### Model testing

The three predictive models were trained with full training sets (i.e. 80% of full set of articles) using the parameters previously described. These models were evaluated for their performance against the remaining 20% of articles in the test set. The accepted and rejected article ratios in the training set and test set reflect that of the dbPEC and dbPTB.

### Model evaluation

The predictive models were evaluated based on their ‘gene recall.’ Gene recall is defined as the percentage of curator-accepted genes found in the original articles that were extracted from articles correctly labeled as ‘considered’ by the classifier. We estimated that a reasonable threshold for our purposes is a gene recall of 95%. To achieve this threshold, articles are classified as ‘considered’ if they have predictive probability greater than or equal to a predicative probability for which 95% of the genes associated with the disease from the test set are recovered. This is sufficient for classifying the vast majority of genes related to a condition of interest using published literature. We used ‘F-gene score’ as an additional metric for classifier performance. ‘F-gene score’ is defined as the harmonic average of gene recall and precision, and can take on values between 0 and 1. ‘F-gene score’ was used to weigh the percentage of curator-accepted genes captured with the percentage of correctly labeled positive papers.

The performance of our predictive models was assessed using recall, precision, gene recall, workload saving, F_1_ score and F-gene score (Table 1). Workload saving is the proportion of articles that our model labeled ‘not considered’ out of the total number of articles. As such, it measures the amount of work that can be saved for future curators (22). A desirable workload saving approaches the percentage of articles ‘not considered’ by manual curation. As a metric for evaluating the accuracy of our model, Area Under the Receiver Operator Characteristic (AUROC) was calculated. AUROC are widely used and packages exist which compare ROC curves and generate *P* values. Model performance between ‘Bag-of-Words’ and the other feature sets was compared using pROC. pROC is a package written in R that compares the AUC for ROC curves (34) with the DeLong method, which utilizes U-statistics and asymptotic normality in order to compare ROC curves without the use of bootstrapping. In addition, the area under the precision-recall curve (AUCPR) was calculated using the R package PRROC (35). Precision-recall curves do not take into account true negatives; therefore, AUCPR is not skewed by an imbalance in the number of positive and negative articles and is typically used for evaluating imbalanced test sets. *P* values could not be calculated for AUCPR and so 5-fold cross-validation was utilized to provide confidence intervals for the AUCs.

**Table 1:** Metrics used to assess performance of predictive models. ^*^G^TP^ is the number of unique curator-accepted genes in true positive articles. G^(TP + FN)^ is the number of unique curator-accepted genes in true positive (TP) or false negative (FN) articles

**Metric**	**Equation**
**Recall**	TP/(TP + FN)
**Precision**	TP/(TP + FP)
**Gene Recall**	^*^G^TP^/G^(TP + FN)^
**Workload Saving**	(TN + FN)/(TP + FP + TN + FN)
**F-gene Score**	2 (Gene-Recall ^*^Precision) / (Precision + Gene-Recall)
**F**_**1**_ **Score**	2 (Recall ^*^Precision) / (precision + recall)

## Code availability

The methods described in this study were implemented largely using the Julia Programming Language, including for building feature sets, training and testing the classifiers. The programs are available from Zenodo (https://doi.org/10.5281/zenodo.3376769) and freely available for public use.

## Results

### Class imbalance

We found no significant differences (pROC, *P* > 0.05) in the AUROC curves for all methods addressing class imbalances for dbPEC. Training and testing on dbPTB showed a significant increase (pROC, *P* < 0.05) in the AUROC of the Neural Network classifier between models with class imbalances addressed by weights when compared to oversampling shown in Supplementary Table S2 and Supplementary Table S3. Across all classifiers trained and tested on dbPEC, the methods for dealing with class imbalances were shown to have similar AUCPRs shown in Supplementary Table S2 and Supplementary Table S3. In addition, for all classifiers trained and tested on dbPTB, all methods for dealing with class imbalances were shown to have similar AUCPRs. For all subsequent analysis, class imbalances were managed by assigning class weights.

### Parameter optimization

Hyper-parameters for the Logistic Regression classifier were optimized using random search 3-fold cross-validation over 1000 iterations. The optimized value for max_iter was determined to be 155 and the optimized value for C was determined to be 1.0 using ‘sag’ as the optimization algorithm. After optimization for the Random Forest classifier, max_features was determined to be 0.8, min_sample_split was determined to be 3, min_impurity_decrease was determined to be 0.0066423, min_sample_leaf was determined to be 4, and criterion was determined to be ‘entropy.’ Optimization for Neural Networks was determined for the four hyper-parameters alpha, learning_rate_init, hidden_layers_sizes, and learning_rate and optimized to be 0.0029, 0.0139, (60160), and ‘invscaling’ respectively. Hyperparamaters and their optimized value are shown in Supplementary Table S4.

### Model testing

To test relative contributions of each of the features, features were tested individually, as well in combination. The feature sets used and the size of each of the feature sets are shown in Table 2. Groupings of feature sets were chosen based on feature size and individual feature performance. S1 contains all features except for ‘Bag-of-Words’, testing how the all the features work in conjunction. S2 contains all the features in S1 except for MeSH, since MeSH is the largest feature set and is the most computationally expensive feature to run. S3 contains all the features in S2 except Gene Count, as gene count was one of the worst performing individual feature sets. S4 contains all features in S1 except for Gene-Subject-Predicate. S5 contains all features in S1 except for Gene Count. S6 contains all the features in S1 except for both Gene-Subject-Predicate and Gene Count.

**Table 2:** Feature set names. A ‘–‘in the Features in Set column denotes that the Features in Set is the same as the set name. The size of the feature set for both dbPEC and dbPTB are listed

**Set Name**	**Features in Set**	**dbPEC**	**dbPTB**
**MeSH**	–	11 467	11 157
**Gene- Significance**	–	1462	1498
**Gene-Subject-Predicate**	–	56	52
**Semtype Count**	–	390	375
**Species Check**	–	1177	1413
**Gene Count**	–	6	6
**Bag-of-Words**	–	34 494	31 926
**S1**	MeSH + Gene Significance + Semtype Count + Gene-Subject-Predicate + Species Check + Gene Count	14 558	14 501
**S2**	Gene Significance + Semtype Count + Gene-Subject-Predicate + Species Check + Gene Count	3091	3344
**S3**	Gene Significance + Semtype Count + Gene-Subject-Predicate + Species Check	3085	338
**S4**	MeSH + Gene Significance + Semtype Count + Species Check + Gene Count	14 502	14 449
**S5**	MeSH + Gene Significance + Semtype Count + Gene-Subject-Predicate + Species Check	14 552	14 495
**S6**	MeSH + Gene Significance + Semtype Count + Species Check	14 496	14 443

The AUROC ranged from 0.901 to 0.920 using logistic regression, from 0.857 to 0.922 using random forests and from 0.869 to 0.894 using the neural network for the dbPTB data set (Table 3). S1 showed a significant increase (pROC, *P* < 0.005) in AUC for the Random Forests classifier trained and tested on dbPTB when compared to ‘Bag-of-Words’. For the dbPEC data set, the AUROC for the different predictive models ranged from 0.743 to 0.805 using logistic regression, from 0.761 to 0.837 using random forests and from 0.689 to 0.782 using neural networks (Table 4). S1 showed no significant (pROC, *P* > 0.05) differences compared to ‘Bag-of-Words’ for any of the classifiers. AUCPR ranged from 0.572 to 0.646 using logistic regression, from 0.616 to 0.680 using random forests and from 0.509 to 0.643 using the neural network for the dbPTB data set (Table 3). For the dbPEC data set, the AUCPR for the different predictive models ranged from 0.597 to 0.653 using logistic regression, from 0.623 to 0.678 using random forests and from 0.531 to 0.619 using neural networks (Table 4). When using the random forests and neural network classifiers trained and tested on both PTB and PEC, S1 showed a greater AUCPR when compared to the other feature sets; however, this difference is within the 95% confidence interval.

**Table 3:** AUC of the ROC curve for all features trained and tested on dbPTB. 5-fold cross-validation was used to determine average AUCPR with a 95% confidence interval. *P*-values are listed for each feature set comparing ROC curves to the bag-of-words ROC curves using pROC. A ‘–‘was used to denote bag-of-words being compared to itself

	**Feature Set**	**AUCPR**	**ROC**	
			**AUC**	***P*-value**
**Logistic Regression**	**BOW**	0.646 ± 0.031	0.906	-
	**S1**	0.622 ± 0.063	0.908	0.918
	**S2**	0.572 ± 0.078	0.919	0.572
	**S3**	0.572 ± 0.066	0.920	0.505
	**S4**	0.619 ± 0.064	0.919	0.526
	**S5**	0.630 ± 0.048	0.901	0.825
	**S6**	0.626 ± 0.045	0.907	0.974
**Random Forests**	**BOW**	0.621 ± 0.040	0.826	–
	**S1**	0.680 ± 0.077	0.922	0.002
	**S2**	0.628 ± 0.070	0.872	0.137
	**S3**	0.616 ± 0.069	0.862	0.251
	**S4**	0.678 ± 0.093	0.865	0.221
	**S5**	0.673 ± 0.077	0.857	0.331
	**S6**	0.667 ± 0.083	0.870	0.155
**Neural Networks**	**BOW**	0.631 ± 0.045	0.894	–
	**S1**	0.643 ± 0.022	0.893	0.979
	**S2**	0.530 ± 0.059	0.872	0.488
	**S3**	0.509 ± 0.075	0.884	0.755
	**S4**	0.599 ± 0.031	0.869	0.393
	**S5**	0.580 ± 0.048	0.891	0.892
	**S6**	0.607 ± 0.098	0.892	0.923

**Table 4:** AUC of the ROC curve and AUC of the precision-recall curve for all features trained and tested on dbPEC. 5-fold cross-validation was used to determine average AUCPR with a 95% confidence interval. *P*-values are listed for each feature set comparing ROC curves to the bag-of-words ROC curves using pROC. A ‘–‘was used to denote bag-of-words being compared to itself

	**Feature Set**	**AUCPR**	**ROC**	
			**AUC**	***P*-value**
**Logistic Regression**	**BOW**	0.653 ± 0.043	0.802	–
	**S1**	0.652 ± 0.028	0.805	0.875
	**S2**	0.600 ± 0.027	0.746	0.007
	**S3**	0.597 ± 0.027	0.743	0.005
	**S4**	0.642 ± 0.027	0.805	0.854
	**S5**	0.639 ± 0.028	0.801	0.991
	**S6**	0.633 ± 0.066	0.802	0.985
**Random Forests**	**BOW**	0.651 ± 0.041	0.837	–
	**S1**	0.678 ± 0.023	0.805	0.064
	**S2**	0.636 ± 0.033	0.777	0.001
	**S3**	0.623 ± 0.043	0.761	0
	**S4**	0.673 ± 0.031	0.802	0.043
	**S5**	0.664 ± 0.038	0.806	0.065
	**S6**	0.641 ± 0.090	0.804	0.053
**Neural Networks**	**BOW**	0.613 ± 0.027	0.763	–
	**S1**	0.619 ± 0.052	0.782	0.372
	**S2**	0.531 ± 0.037	0.689	0.007
	**S3**	0.554 ± 0.038	0.691	0.009
	**S4**	0.595 ± 0.051	0.764	0.946
	**S5**	0.598 ± 0.048	0.771	0.696
	**S6**	0.610 ± 0.003	0.743	0.417

### Model evaluation

The performance of the models using the three classifiers and various feature sets is shown in Tables 5 and 6. For dbPTB, workload savings at a 95% gene recall threshold ranged from 0.797 with Random Forest to 0.814 with Neural Networks, compared to the actual manual rejection rate 0.846. For dbPEC, workload savings at a 95% gene recall threshold for dbPEC ranged from 0.283 with Neural Networks to 0.371 with Random Forests, compared to the actual manual rejection rate for of 0.492. In addition, all features sets, except S3 and S4, outperformed ‘Bag-of-Words’ in terms of workload savings on every classifier for both dbPTB and dbPEC. Notably S1, S5 and S6 showed the best performance at a 95% gene recall. In addition, S1, S5 and S6 outperformed ‘Bag-of-Words’ on every classifier in terms of both F_1_ score and F-gene score, for both dbPEC and dbPTB.

**Table 5:** The values of the performance metrics for each feature set trained and tested on dbPTB. Performance metrics were recorded for each classifier and values were recorded at a 95% gene Recall threshold

	**Feature Set**	**Recall**	**Gene Recall**	**Precision**	**F Score**	**F-gene Score**	**Workload Savings**
**Logistic Regression**	**BOW**	0.805	0.956	0.379	0.516	0.543	0.716
	**S1**	0.829	0.956	0.531	0.648	0.683	0.791
	**S2**	0.756	0.956	0.544	0.633	0.693	0.814
	**S3**	0.829	0.956	0.523	0.642	0.676	0.788
	**S4**	0.756	0.956	0.534	0.626	0.686	0.810
	**S5**	0.805	0.956	0.541	0.647	0.691	0.801
	**S6**	0.854	0.956	0.556	0.673	0.703	0.794
**Random Forests**	**BOW**	0.829	0.965	0.262	0.398	0.412	0.575
	**S1**	0.878	0.956	0.379	0.529	0.543	0.690
	**S2**	0.707	0.956	0.460	0.558	0.621	0.794
	**S3**	0.683	0.956	0.406	0.509	0.570	0.775
	**S4**	0.683	0.956	0.452	0.544	0.613	0.797
	**S5**	0.659	0.956	0.435	0.524	0.598	0.797
	**S6**	0.659	0.956	0.429	0.519	0.592	0.794
**Neural Networks**	**BOW**	0.829	0.956	0.374	0.515	0.537	0.703
	**S1**	0.707	0.956	0.617	0.659	0.750	0.846
	**S2**	0.805	0.956	0.429	0.559	0.592	0.748
	**S3**	0.756	0.956	0.443	0.559	0.605	0.771
	**S4**	0.756	0.956	0.508	0.608	0.664	0.801
	**S5**	0.756	0.956	0.544	0.633	0.693	0.814
	**S6**	0.756	0.956	0.554	0.639	0.701	0.817

**Table 6:** The values of the performance metrics for each feature set trained and tested on dbPEC. Performance metrics were recorded for each classifier and values were recorded at a 95% gene Recall threshold

	**Feature Set**	**Recall**	**Gene Recall**	**Precision**	**F Score**	**F-gene Score**	**Workload Savings**
**Logistic Regression**	**BOW**	0.950	0.951	0.425	0.588	0.588	0.247
	**S1**	0.967	0.956	0.439	0.604	0.602	0.258
	**S2**	0.939	0.951	0.421	0.582	0.584	0.249
	**S3**	0.944	0.966	0.413	0.574	0.578	0.228
	**S4**	0.967	0.956	0.444	0.608	0.606	0.266
	**S5**	0.961	0.951	0.458	0.620	0.618	0.292
	**S6**	0.956	0.951	0.461	0.622	0.621	0.301
**Random Forests**	**BOW**	0.939	0.956	0.448	0.607	0.610	0.294
	**S1**	0.928	0.951	0.488	0.640	0.645	0.360
	**S2**	0.922	0.956	0.445	0.600	0.607	0.301
	**S3**	0.917	0.951	0.426	0.582	0.589	0.275
	**S4**	0.950	0.951	0.491	0.648	0.648	0.348
	**S5**	0.922	0.961	0.494	0.643	0.653	0.371
	**S6**	0.922	0.956	0.484	0.635	0.643	0.358
**Neural Networks**	**BOW**	0.967	0.956	0.407	0.572	0.570	0.199
	**S1**	0.961	0.966	0.410	0.575	0.576	0.210
	**S2**	0.922	0.951	0.400	0.558	0.563	0.223
	**S3**	0.944	0.951	0.369	0.530	0.531	0.137
	**S4**	0.967	0.966	0.371	0.536	0.536	0.122
	**S5**	0.961	0.956	0.448	0.611	0.610	0.277
	**S6**	0.922	0.956	0.433	0.590	0.596	0.283

## Discussion

This study explored the potential of using machine learning approaches to identify scientific articles with genes or genetic information relevant to complex diseases. We used logistic regression, random forests, and neural networks to classify articles relevant to the diseases of interest that should be considered for further formal analysis. Random search cross-validation was used to optimize for the hyper-parameters of the various classifiers. This method was used instead of grid search cross-validation due to the former being less computationally demanding (31). Our previously published and publicly accessible, curated databases for two complex diseases, preterm birth and preeclampsia, served as our reference data sets. To test the models, articles were separated into a training and test set. Given the complexity of this classification task, 80% of the articles were selected for the training set to ensure that we had a sufficient number of training examples to develop a reliable predictive model. This 80% training set approach comports with the training set size used in an example evaluation of the Scikit-learn’s liblinear logistic regression (36). Class imbalances can adversely influence classifier performance due to predictive bias in favor of the majority class (17). Since the majority class is more heavily represented in the dataset, it tends to have more influence on cases of uncertainty, which can lead to over prediction of majority cases (17). Using pROC, ROC curves for the various methods for dealing with class imbalances were compared. No significant differences (pROC, *P* > 0.05) were found between various methods for dealing with class imbalances in dbPEC. In dbPTB, weights were determined to significantly increase AUROC for the neural network classifier. For both dbPTB and dbPEC, AUCPR was found to be similar across all classifiers, for all methods of dealing with class imbalances. As such, class imbalances were managed by assigning class weights.

We compared the performance of each of the machine learning classifiers trained with combinations of different feature sets. The purpose of our approach was primarily to reduce the total number of articles that we needed to review manually in order to identify the genes associated with a condition of interest. This meant that prioritizing a predictive model that classifies articles with high recall was important. However, recall alone is not entirely adequate for evaluating the ability of a classifier to help curators identify the genes associated with pathogenesis. Instead, we defined two new measures (gene recall and F-gene score) for evaluation of the classifiers. In addition, we defined novel feature sets to identify species, gene mentions, gene-subject interactions, and gene-quantitative concept co-occurrences in the titles and abstracts. These feature sets were tested independently and in various combinations. The combined feature sets S1, S2, S5 and S6 showed greater degrees of workload savings across all classifiers when compared to ‘Bag-of-Words.’ In addition, these feature sets were much smaller than ‘Bag-of-Words’, making them more computationally inexpensive and better for curating large quantities of articles. Our results suggest that machine learning algorithms can identify articles of interest for creation or maintenance of a database or gene set for complex diseases. Given the enormity of the manual classifications of articles reviewed for the reference databases, we conclude that our pipeline performed well for its ability to both prioritize articles with relevant genetic information and decrease curator workload. Taken together, our analysis shows that automation can help curators more efficiently review literature for genetic markers of human disease while still maintaining accuracy comparable to strict manual curation.

In the developed pipeline, we used several text-mining tools to annotate and extraction information from the title and abstract of articles. The rich feature-set gathered from annotated titles and abstracts using these tools allowed us to develop a predictive model that met our standards for gene recall and provided reasonable workload savings. The workload savings, while markedly different between our analysis of preterm birth and preeclampsia data sets, were reasonably close to the percentage of papers that were ‘not considered’ by manual curation. These savings are sufficiently large to justify use of our pipeline in future curation efforts and maintenance of these databases. Furthermore, with a gene recall of 95%, our model captures most relevant genes. Genes not captured are likely to be identified by other means, such as the screening of publicly available databases for genetic data or pathway-based gene imputation (37).

When assessing the performance of our pipeline, we acknowledge the abundance of similar tools that utilize machine learning to classify articles as relevant or irrelevant for curation. Many such tools have been developed to simplify systematic review. Notable examples include AbstrackR (13) and Rayyan (12). These tools and others have been shown to classify articles for inclusion in systematic review with recall that outperform our models (11, 22). For our data sets, at a 95% gene recall threshold, AbstrackR yielded a greater recall but lower workload savings and precision, recovering more articles with less genetic relevance [data not shown]. Although these tools may be useful for automating triage for many Systematic Reviews, for a more nuanced curation task, such as identifying articles to maintain a phenotype-specific genetic database, it may be helpful to utilize a more specialized machine learning approach as our own.

Beyond what has already been described, a significant advantage of our pipeline is that it allows for granular control over classification, with the added benefit of generating a MySQL database that stores relevant article information relevant to curation teams. This includes descriptive metadata and the annotations previously described. Having this easily accessible data enables curation teams to further characterize the set of ‘considered’ articles and information useful for future association testing. With our pipeline, tasks such as the identification of all genes and mutations mentioned in the titles and abstracts can be performed in a single query. Given that our pipeline is specifically designed to identify articles with relevant genetic information, it may be the preferred approach for those curating literature for the genetic study of complex diseases.

A possible limitation of our approach is that it is unclear how well our pipeline will perform on other data sets. Our separate evaluations of dbPTB and dbPEC reveal different values for recall, specificity, gene recall and workload reduction. This variance is likely multifactorial in origin. The accuracy of our predictive model will be dependent on how articles were originally selected for consideration, how robust the collection of literature is on a given condition of interest, the number of genes that have been shown to contribute to the condition, and variations in how the condition is characterized in biomedical literature. Using this pipeline to curate articles relevant for the genetic study of other conditions will be necessary for further evaluation.

Additional features and classifiers were evaluated that were not used in our final pipeline. Other classifiers that were considered included a support vector machine and a second neural network. The support vector machine was omitted due to inferior performance as well as known but minor inaccuracies in its probabilistic output (4). A second multilayer perceptron was also developed using Mocha.jl (https://github.com/pluskid/Mocha.jl), but it was omitted due to poor performance using Mocha.jl. Additional features that were considered included the journal ISSN and publication year. The journal ISSN associated with each article was not used because it did not improve prediction accuracy. Publication year was not used due to bias in the training set, as the preeclampsia data set only included articles published in 2014 and 2015 that were rejected during manual curation. Accepted papers published in 2014 and 2015 had not been updated in the preeclampsia database at the time of data access.

## Conclusions

We have developed a machine learning-based computational pipeline that can identify of articles that meet criteria for formal curation. This approach allows for a significant reduction in curation workload for those seeking a comprehensive collection of literature that documents the genes related to a phenotype of interest. This approach may prove to be generalizable to other phenotypes or diseases of interest with a robust base of publications. Furthermore, comparative evaluation of the machine learning models demonstrated that the combined feature sets S1, S2, S5 and S6 performed better in terms of workload savings than bag-of-words. In addition, S1, S5 and S6 were shown to outperform bag-of-words in F_1_ score and F-gene score. Moreover, our feature sets are less than half the size of bag-of-words and as such are less computationally expensive. This is notable particularly when curating large quantities of articles. Use of these predictive models can potentially improve the efficiency of future curation efforts.

## Supplementary Material

Supplementary_Tables_baz124
