# Supplementary material for: Machine learning approach to literature mining for the genetics of complex diseases
Source: Database (Oxford). 2019 Nov 26;2019:baz124. doi: 10.1093/database/baz124 (PMC6877776; doi:10.1093/database/baz124)
Supplement: Supplementary_Tables_baz124 [file supplementary_tables_baz124.docx]

**Supplementary Table 1. List of queries for searching related articles for two databases, dbPEC and dbPTB using SciMiner.**

|  | |
| --- | --- |
| **Databases** | **Queries** |
| **dbPEC** | Preeclampsia AND Genetics |
|  | Pre-eclampsia and Genetics |
|  | Pre-eclampsia AND gene expression analysis |
|  | Preeclampsia AND gene expression analysis |
|  | Preeclampsia AND RNA |
|  | Pre-eclampsia AND RNA |
|  | Preeclampsia AND gene |
|  | Pre-eclampsia AND gene |
|  | Preeclampsia AND protein expression |
|  | Pre-eclampsia AND protein expression |
|  | HELLP AND genetics |
| **dbPTB** | Preterm birth AND genes |
|  | Preterm birth AND pedigree |
|  | Preterm birth AND single nucleotide polymorphism |
|  | Preterm birth AND inheritance |
|  | Preterm birth AND gene frequency |
|  | Preterm birth AND genetic predisposition |
|  | Preterm birth AND birth timing |
|  | Preterm birth AND genotype |
|  | Preterm birth AND prematurity rate |

**Supplementary Table 2. AUC of the ROC and AUC of the Precision-Recall Curve for the different methods for dealing with class imbalance, trained and tested on dbPTB. 5-fold cross-validation was used to determine average AUCPR with a 95% confidence interval. p-values were listed for each method comparing ROCs to Weights ROC using pROC. A ‘-‘ was used to denote Weights being compared to itself.**

|  | **Sampling Method** | **AUCPR** | **ROC** | |
| --- | --- | --- | --- | --- |
|  |  |  | **AUC** | **P-value** |
| **Logistic Regression** | **Weights** | 0.531 ± 0.092 | 0.731 | - |
|  | **Oversampling** | 0.548 ± 0.036 | 0.739 | 0.080 |
|  | **Undersampling** | 0.518 ± 0.078 | 0.749 | 0.550 |
|  | **Oversampling + Undersampling** | 0.592 ± 0.097 | 0.761 | 0.332 |
| **Random Forests** | **Weights** | 0.602 ± 0.072 | 0.748 | - |
|  | **Oversampling** | 0.551 ± 0.053 | 0.740 | 0.223 |
|  | **Undersampling** | 0.650 ± 0.043 | 0.756 | 0.771 |
|  | **Oversampling + Undersampling** | 0.648 ± 0.069 | 0.763 | 0.603 |
| **Neural Networks** | **Weights** | 0.466 ± 0.070 | 0.674 | - |
|  | **Oversampling** | 0.466 ± 0.097 | 0.675 | 0.952 |
|  | **Undersampling** | 0.461 ± 0.116 | 0.682 | 0.810 |
|  | **Oversampling + Undersampling** | 0.449 ± 0.091 | 0.698 | 0.476 |

**Supplementary Table 3. AUC of the ROC and AUC of the Precision-Recall Curve for the different methods for dealing with class imbalance, trained and tested on dbPEC. 5-fold cross-validation was used to determine average AUCPR with a 95% confidence interval. p-values were listed for each method comparing ROCs to Weights ROC using pROC. A ‘-‘ was used to denote Weights being compared to itself.**

|  | **Sampling Method** | **AUCPR** | **ROC** | |
| --- | --- | --- | --- | --- |
|  |  |  | **AUC** | **P-value** |
| **Logistic Regression** | **Weights** | 0.577 ± 0.020 | 0.871 | - |
|  | **Oversampling** | 0.587 ± 0.056 | 0.879 | 0.197 |
|  | **Undersampling** | 0.573 ± 0.048 | 0.878 | 0.881 |
|  | **Oversampling + Undersampling** | 0.598 ± 0.047 | 0.892 | 0.607 |
| **Random Forests** | **Weights** | 0.599 ± 0.040 | 0.842 | - |
|  | **Oversampling** | 0.634 ± 0.038 | 0.850 | 0.570 |
|  | **Undersampling** | 0.625 ± 0.035 | 0.879 | 0.429 |
|  | **Oversampling + Undersampling** | 0.633 ± 0.032 | 0.873 | 0.497 |
| **Neural Networks** | **Weights** | 0.525 ± 0.041 | 0.828 | - |
|  | **Oversampling** | 0.521 ± 0.017 | 0.802 | 0.031 |
|  | **Undersampling** | 0.524 ± 0.025 | 0.812 | 0.429 |
|  | **Oversampling + Undersampling** | 0.555 ± 0.057 | 0.828 | 0.991 |

**Supplementary Table 4. Hyper-parameters of the various classifiers and their optimized value. Hyper-parameters were optimized for AUC.**

|  | **Hyperparameter** | **Optimized Value** |
| --- | --- | --- |
|  | max_iter | 155 |
| **Logistic Regression** | C | 1 |
|  | solver | "sag" |
|  | max_features | 0.8 |
|  | min_sample_split | 3 |
| **Random Forests** | min_impurity_decrease | 0.00664243 |
|  | min_samples_leaf | 4 |
|  | criterion | "entropy" |
|  | learning_rate | "invscaling" |
| **Neural Networks** | alpha | 0.0029 |
|  | learning_rate_init | 0.0139 |
|  | hidden_layer_sizes | (60,160) |
